# Crystal structure, solvothermal synthesis, thermogravimetric studies and DFT calculations of a five-coordinate cobalt(II) compound based on the *N*,*N*-bis­(2-hy­droxy­eth­yl)glycine anion

**DOI:** 10.1107/S2056989016014596

**Published:** 2016-09-23

**Authors:** Yanling Zhou, Xianrong Liu, Qijun Wang, Lisheng Wang, Baoling Song

**Affiliations:** a School of Chemistry and Chemical Engineering, Guangxi University, Nanning, 530004, People’s Republic of China; b Guangxi Experiment Centre of Science and Technology, Guangxi University, Nanning, 530004, People’s Republic of China

**Keywords:** Five-coordinate transition metal, crystal structure, solvothermal synthesis, mononuclear, ESI–MS, DFT

## Abstract

The reaction of CoCl_2_·6H_2_O, *N*,*N*-bis­(2-hy­droxy­eth­yl)glycine and tri­ethyl­amine (Et_3_N) in ethanol solution under solvothermal conditions produced crystals of [*N*,*N*-bis­(2-hy­droxy­eth­yl)glycinato]chloridocobalt(II), [Co(C_6_H_12_NO_4_)Cl]. The Co^II^ ion is coordinated in a slightly distorted trigonal–bipyramidal environment which is defined by three O atoms occupying the equatorial plane and the N and Cl atoms in the apical sites. In the crystal, two types of O—H⋯O hydrogen bonds connect the mol­ecules, forming a two-dimensional network parallel to (001). The mol­ecular structure of the title compound confirms the findings of FTIR, elemental analysis, ESI–MS analysis and TG analysis. By using the density functional theory (DFT) (B3LYP) method with 6-31G(d) basis set, the molecular structure has been calculated and optimized.

## Abstract 2

The mol­ecular structure of the title compound confirms the findings of FTIR, elemental analysis, ESI–MS analysis and TG analysis. By using the density functional theory (DFT) (B3LYP) method with 6-31G(d) basis set, the structure has been calculated and optimized. In the crystal, two types of O—H⋯O hydrogen bonds connect the mol­ecules, forming a two-dimensional network parallel to (001).

## Chemical context   

In recent years, coordination compounds have attracted a great deal of inter­est for their structural aesthetics and potential functional applications (Fujita *et al.*, 2004 ▸). The design of mol­ecular structures is highly influenced by factors such as the coordination nature of the metal ion, the coordin­ating ability and functionality of the organic ligands and the reaction conditions (Zhang *et al.*, 2015 ▸; Yin *et al.*, 2015 ▸). Hence, the prediction of crystal structure is largely considered to be serendipitous except for simple compounds such as mononuclear mol­ecules. The 3*d* ^7^configuration of Co^II^ is particularly suited for the construction of metal–organic compounds (Kurmoo, 2009 ▸). One of the inter­esting structural aspects of studying cobalt compared to nickel, iron or manganese is the range of coordination geometries – octa­hedral, tetra­hedral, square–pyramidal, trigonal–bipyramidal and square–planar – which are all stable (Kurmoo, 2009 ▸). There are several coordination modes for the cobalt ion. The common mode is six-coordinate (Bryant *et al.*, 2015 ▸; Artetxe *et al.*, 2015 ▸), and only relatively few four-coordinate (Gupta *et al.*, 2015 ▸) and five-coordinate (Lee *et al.*, 2015 ▸) cobalt complexes have been recorded. Generally, five-coordinate compounds have two classical configurations, trigonal–bipyramidal and square–pyramidal, and the extent of each geometry each can be determined by the τ value (Addison *et al.*, 1984 ▸).

**Figure 1:**
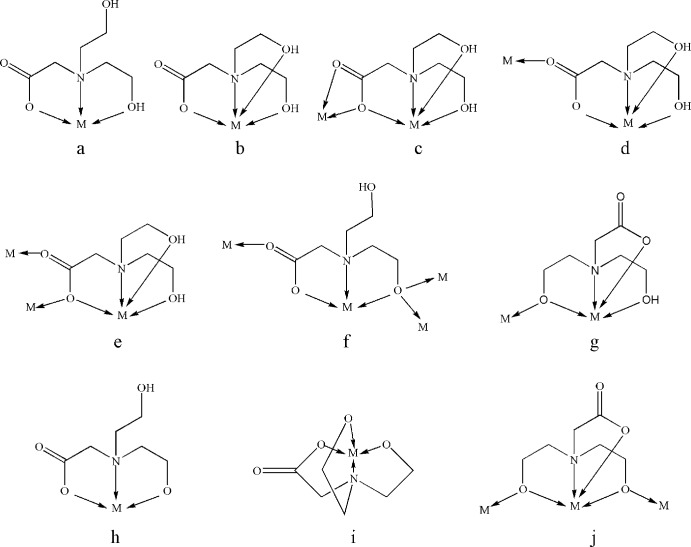
Coordination modes for bicH_2_^−^ (a–e), bicH^2−^ (f–h) and bic^3−^ (i, j).

The carboxyl­ate unit is widely used in the synthesis of coordination compounds and is part of commonly used ligands. It is a good bridging group, favouring the formation of products (Zhou *et al.*, 2009 ▸). Very recently, we have been investigating Co^II^ compounds constructed from ligands containing carboxyl­ate and hydroxyl groups, which usually form multinuclear and/or polymeric structures and show inter­esting magnetic behavior (Zhou *et al.*, 2009 ▸; Zeng *et al.*, 2010 ▸). Similarly, herein, we chose *N*,*N*-bis­(2-hy­droxy­eth­yl)glycine (bicH_3_) containing two hydroxyl oxygen atoms, one carboxylate oxygen atom and one nitro­gen atom, which can potentially coordinate to a metal ion as a tetra­dentate ligand (He *et al.*, 1999 ▸). BicH_3_ contains the properties of both amino acid and amino alcohol as a result of the *N*-substituted amino, carboxyl, and two hydroxyl groups in the mol­ecule. To the best of our knowledge, the crystal structures of metal–organic compounds with the bicH_3_ ligand have not been very well explored to date. Potential coordination modes for bicH_2_^−^, bicH^2−^ and bic^3−^ are shown in Fig. 1 ▸. In the course of our ongoing studies on Co^II^ compounds containing ligands with carboxylate moieties, we have directly assembled the title compound [Co(bicH_2_)Cl], **1**, using the flexible tetra­dentate ligand bicH_3_ and CoCl_2_·6H_2_O under solvothermal conditions.
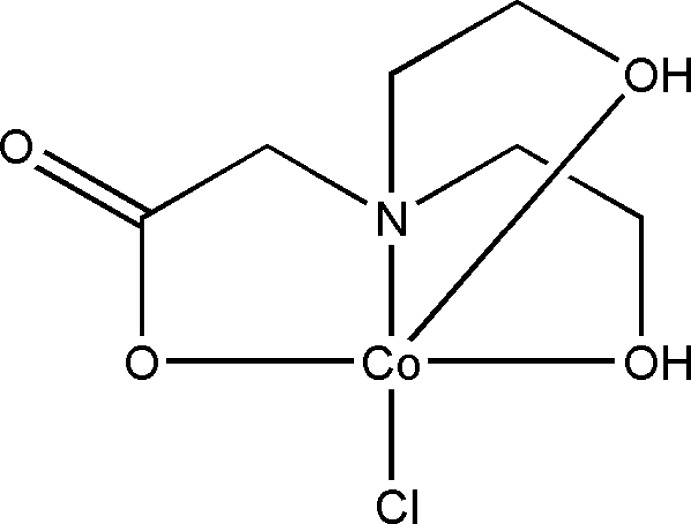


## Structural commentary   

**Figure 2:**
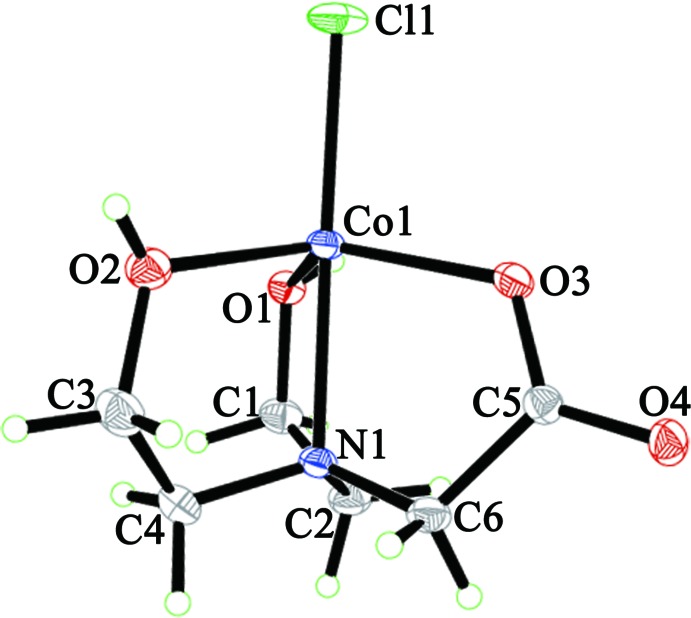
The mol­ecular structure of the title compound, showing the atom labeling. Displacement ellipsoids are drawn at the 30% probability level.

The mol­ecular structure of the title compound is shown in Fig. 2 ▸. The Co^II^ ion is five-coordinated by four atoms from the bicH_2_^−^ ligand (one carboxyl­ato oxygen atom, two hydroxyl oxygen atoms, one nitro­gen atom) and one terminal chlorine atom in a slightly distorted trigonal–bipyramidal environment (*τ* = 0.94, τ = |α − β|/60, α and β being the two largest angles around the central atom; values for τ in perfect coordination geometries are 1.0 for trigonal–bipyramid and 0.0 for square–pyramidal). In a similar reported compound which was formed by bicH_2_^2−^ and a Cu^II^ ion, a five-coordinate mode was observed (He *et al.*, 1999 ▸); the difference is that one nitro­gen atom of benzimidazole or iso­quinoline has replaced the terminal chloride ion in compound **1**. In **1**, the bond lengths around the Co^II^ ion are Co1—N1 = 2.1626 (15), Co1—O1 = 2.0482 (13), Co1—O2 = 2.0463 (14), Co1—O3 = 2.0095 (14) and Co1—Cl1 =2.2701 (6) Å. The length of the Co—O(carboxyl­ate) bond is shorter than that of Co—O(hydrox­yl), which may be due to the difference between the electron density of carboxyl­ate oxygen atoms and that of hydroxyl oxygen atoms (He *et al.*, 1999 ▸). According to the total valence–charge balance and the bond lengths, we can conclude that cobalt is in oxidation state +II.

## Supra­molecular features   

**Figure 3:**
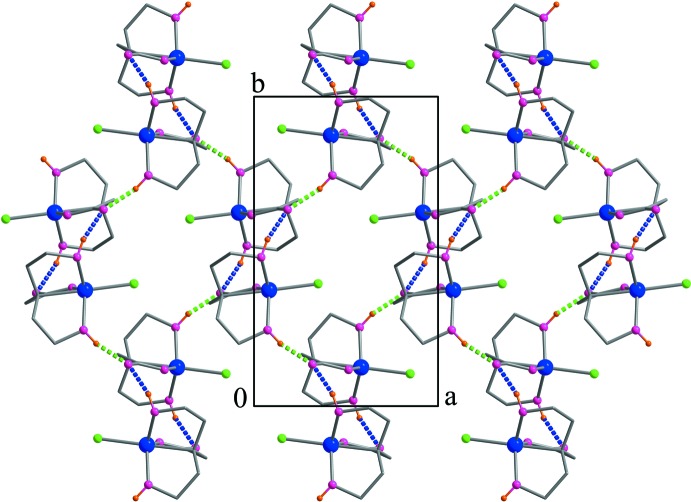
Part of the crystal structure showing the two different O—H⋯O hydrogen bonds as distinct colors, blue for O1—H⋯O4^ii^ bonds and green for O2—H2⋯O4^i^ bonds (symmetry codes as in Table 1 ▸).

In the crystal, two types of O—H⋯O hydrogen bonds (Table 1 ▸) connect the mol­ecules, forming a two-dimensional network parallel to (001) (Fig. 3 ▸). The O—H groups behave as donors to the non-coordinating carboxyl­ate oxygen atom of symmetry-related mol­ecules. The hy­droxy group containing O1 acts as a bifurcated O—H⋯(O,O) donor while caboxylate atom O4 is a bifurcated (O—H,O—H)⋯O acceptor.

## Database survey   

A search of the Cambridge Structural Database (CSD; Groom *et al.*, 2016 ▸) was carried out for structures containing the bicH_3_ ligand. This revealed bicH_3_ coordinating to a lanthanide metal (Inomata *et al.*, 2001 ▸), Cd and Na (Katsoulakou *et al.*, 2011 ▸), Cu, Ni and Zn (Thakuria & Das, 2007 ▸; Liu *et al.*, 2013 ▸; Lo & Ng, 2010 ▸), Re, Mn and Fe (Kirillov *et al.*, 2005 ▸; Sun *et al.*, 1997 ▸; Graham *et al.*, 2009 ▸). A related structure with copper and bromide (Yamaguchi *et al.*, 1991 ▸) shows a very similar mononuclear crystal structure to the title compound. There are only a small number of reports for the ligand coordinating to Co (Funes *et al.*, 2015 ▸; Zhao & Liu, 2010 ▸; Liu *et al.*, 2015 ▸).

## Synthesis and crystallization   

**Figure 4:**
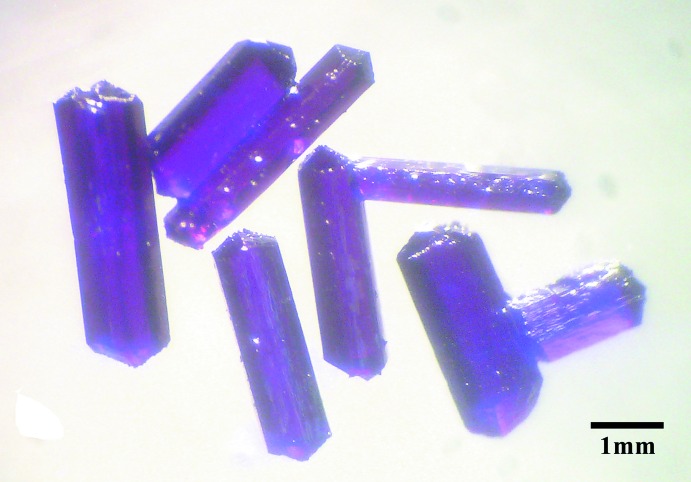
The optical microscope image of single crystals of compound **1**.

The ligand bicH_3_ (0.5 mmol) in a ethanol solution (2 mL) was added to a ethanol solution (5 mL) of CoCl_2_·6H_2_O (1 mmol). 0.02 mL of tri­ethyl­amine was added dropwise to the mixed solution and stirred for 15 min at room temperature. The reactants were sealed in a 12 mL Teflon-lined autoclave, heated at 413 K for three days and then cooled to room temperature at a rate of 10 K h^−1^. Purple single crystals (Fig. 4 ▸) were obtained along with purple powder. The crystals were picked out, washed with distilled water, and dried in air (yield *ca* 50.3% based on Co^II^). Analysis calculated (%) for C_6_H_12_ClCoNO_4_: C 28.30, H 3.93, N 5.50; Found C 28.31, H 3.95, N 5.54%. FTIR data for **1** (KBr, cm^−1^): 3383(*m*), 2964(*w*), 1593(*s*), 1434(*m*), 1407(*m*), 1309(*w*), 1058(*w*), 890(*w*).

## ESI–MS spectroscopic analysis   

**Figure 5:**
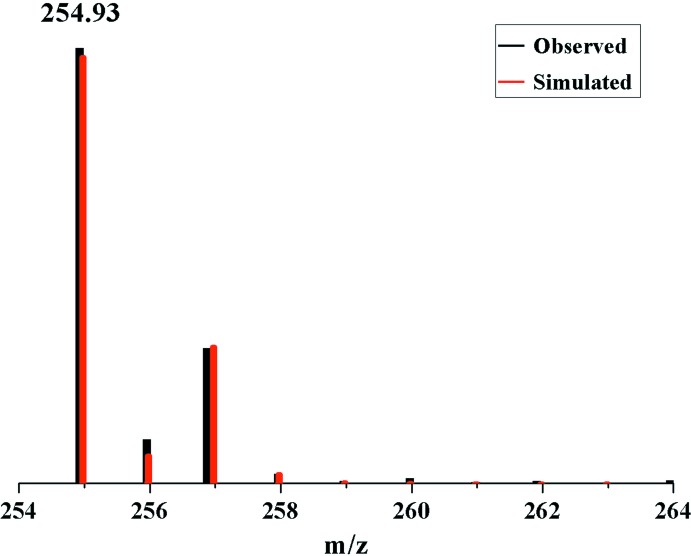
ESI mass spectrum of compound **1**.

The ESI mass spectra were recorded using an LCQ–FLEET mass spectrometer (Thermo). To give further evidence for the inner structure of compound **1**, characterization of the mol­ecule in solution was accomplished by ESI–MS experiments. For the methanol solution of **1**, the ESI mass spectrum (Fig. 5 ▸) exhibits the main ion peak observed at an *m*/*z* of 254.93, which can be assigned as [Co(C_6_H_11_NO_4_)Cl]^+^ (fit: 254.970860). The observed *m*/*z* clearly matches the assigned formula as well as the simulated spectra. This suggests that compound **1** produced in solution was stable during the ionization process. ESI–MS can also be used to examine a series of inner-bridge replacement reactions for multinuclear Co compounds (Zhou *et al.*, 2010 ▸; Hu *et al.*, 2013 ▸), which is an important complement to ligand exchange, ion exchange, template exchange and supra­molecular transformations (Chakrabarty *et al.*, 2011 ▸; Miras *et al.*, 2009 ▸). The study of the chemistry of coordination compounds by mass spectroscopy is an excellent tool to demonstrate the stability and existence of multinuclear moleculesin solution.

## TG analysis   

**Figure 6:**
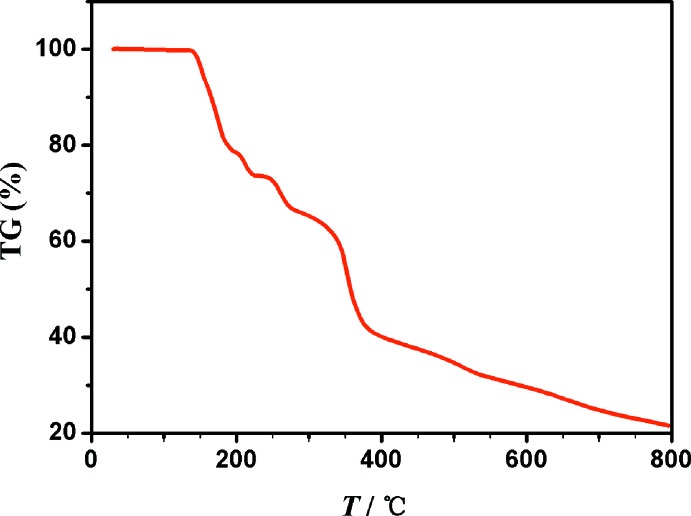
The TG curve of compound **1**.

The TG analysis was performed on Pyris Diamond TG/DTA. The appearance of the flexible polydentate ligands inspired us to investigate the thermal stability of the network. The crushed single-crystal samples were heated to 1073 K in an N_2_ atmosphere at a heating rate of 5 K min^−1^ (Fig. 6 ▸). The TGA curve for **1** shows that the framework begins to decompose at 413 K, and the 21.6% remaining weight is assuming to the mass loss percentage of cobalt (cal. 22.9%). One similar compound, [CuCd(bicH_2_(NO_3_)Cl_2_(H_2_O)]·H_2_O, constructed with bicH_2_ is quite unstable and begins to lose lattice water at 393 K (Liu *et al.*, 2013 ▸). Other compounds synthesized using the same ligand also show mass loss below 413 K (Inomata *et al.*, 2001 ▸), owing to the loss of coordinating water. However, another reported complex with five-coordinate cobalt begins to disintegrate at 669 K (Lee *et al.*, 2015 ▸). In summary, the crystals synthesized using bicH_2_^2−^ tend to decompose at a relatively low temperature.

## DFT calculations   

All the calculations were performed by using the *GAUSSIAN09* program package (Frisch *et al.*, 2009 ▸). The mol­ecular structure of the title compound in the ground state was optimized *in vacuo* without considering the solvent, and a basis set (b1) consisting of a standard LanL2DZ basis set (Dunning & Hay, 1976 ▸; Wadt & Hay, 1985 ▸; Hay & Wadt, 1985 ▸) for Co, while the other atoms, C, H, N, O were described by a standard 6-31G(d) set. To investigate the energy differences between the high-spin and low-spin states of the title compound, the Δ*E* of these two energy states was evaluated using the B3LYP/b1 method (Carabineiro *et al.*, 2008 ▸; Saraçoğlu & Cukurovali, 2016 ▸). Vibrational frequencies were calculated for all stationary points to verify that each was a minimum (NIMAG = 0) on the potential energy surface.

**Figure 7:**
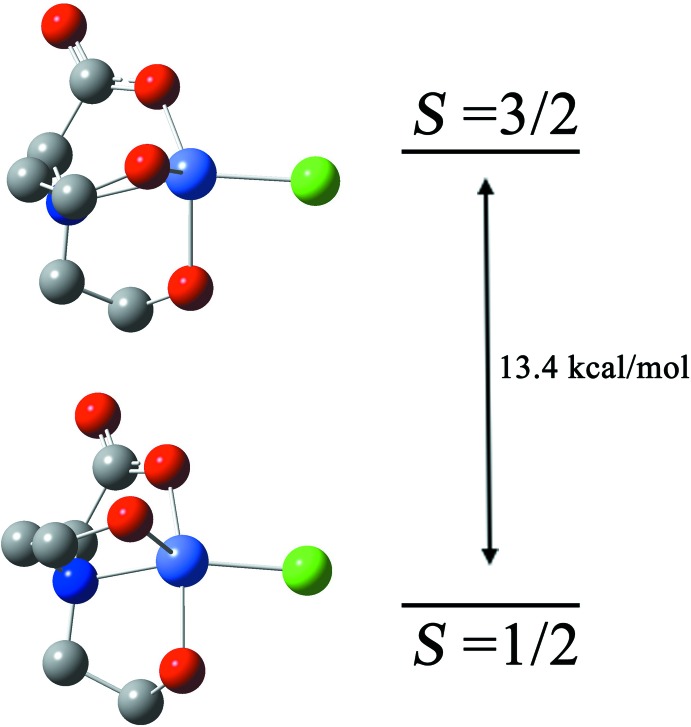
Optimized geometries (B3LYP) for the low-spin (*S* = 1/2, bottom) and the high-spin (*S* = 3/2, top) ground states and the relative energy of the two ground states (kcal mol^−1^).

To gain an insight of the electronic structures, bonding properties and relative stability of the two different spin ground states (*S* = 3/2 and 1/2) for compound **1**, the calculations in the DFT method were investigated. The optimized geometries calculated for compound **1** are presented in Fig. 7 ▸. The structure of **1** presented an almost perfect trigonal–bipyramidal geometry by means of the X-ray diffraction. The chlorine and nitro­gen atoms occupy the axial positions, while the equatorial plane is occupied by three oxygen atoms. According to the energies for the two calculated structures (see Supporting information), the X-ray structure determined for complex **1** should correspond to the complex with high-spin ground state. The result can also be indicated by the mean (δ) and maximum (Δ) absolute deviations obtained for the coordination distances (Co—*X*). For the high-spin form of complex **1**, values of δ = 0.104 Å and Δ = 0.148 Å indicate a reasonable agreement. As for the high-spin ground state and the experimental value, the biggest difference for the bond lengths is found to be 0.148 Å for Co—N, similar to what has been observed in related Co^II^ compounds (Carabineiro *et al.*, 2008 ▸). The results of the schematic representation of both ground states supported its coordination behavior and the value of Δ*E* is 13.4 kcal mol^−1^, which shows that compound **1** can well exist stably. The cartesian coordinates for the two calculated structures are given in the Supporting information.

## Refinement   

Crystal data, data collection and structure refinement details are summarized in Table 2 ▸. H atoms bonded to C atoms were placed in calculated positions with C—H = 0.97 Å and *U*_iso_(H) = 1.2U_eq_(C). Hydroxyl hydrogen atoms H1 and H2 were refined independently, H1 with a refined isotropic displacement parameter and H2 with *U*_iso_(H) = 1.5*U*_eq_(O).

## Supplementary Material

Crystal structure: contains datablock(s) I. DOI: 10.1107/S2056989016014596/lh5821sup1.cif

Structure factors: contains datablock(s) I. DOI: 10.1107/S2056989016014596/lh5821Isup5.hkl

Supporting information file. DOI: 10.1107/S2056989016014596/lh5821sup3.tif

Supporting information file. DOI: 10.1107/S2056989016014596/lh5821sup4.pdf

CCDC reference: 1504322

Additional supporting information: crystallographic information; 3D view; checkCIF report

## Figures and Tables

**Table 1:** Hydrogen-bond geometry (Å, °)

*D*—H⋯*A*	*D*—H	H⋯*A*	*D*⋯*A*	*D*—H⋯*A*
O2—H2⋯O4^i^	0.85 (1)	1.79 (1)	2.6271 (19)	165 (2)
O1—H1⋯O4^ii^	0.79 (3)	1.89 (3)	2.6567 (19)	165 (3)

Symmetry codes: (i) 
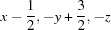
; (ii) 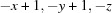.

**Table 2:** Experimental details

Crystal data	
Chemical formula	[Co(C_6_H_12_NO_4_)Cl]
*M*_r_	256.55
Crystal system, space group	Orthorhombic, *P* *b* *c* *a*
Temperature (K)	296
*a*, *b*, *c* (Å)	8.3925 (9), 14.0939 (15), 15.8448 (17)
*V* (Å^3^)	1874.2 (3)
*Z*	8
Radiation type	Mo *K*α
μ (mm^−1^)	2.10
Crystal size (mm)	0.84 × 0.27 × 0.24

Data collection	
Diffractometer	Bruker APEXII CCD
Absorption correction	Multi-scan (*SADABS*; Bruker, 2004 ▸)
*T*_min_, *T*_max_	0.512, 0.604
No. of measured, independent and observed [*I* > 2σ(*I*)] reflections	15130, 1933, 1854
*R*_int_	0.021
(sin θ/λ)_max_ (Å^−1^)	0.625

Refinement	
*R*[*F* ^2^ > 2σ(*F* ^2^)], *wR*(*F* ^2^), *S*	0.025, 0.066, 1.14
No. of reflections	1933
No. of parameters	125
No. of restraints	3
H-atom treatment	H atoms treated by a mixture of independent and constrained refinement
Δρ_max_, Δρ_min_ (e Å^−3^)	0.51, −0.43

Computer programs: *APEX2* and *SAINT* (Bruker, 2004 ▸), *SIR2004* (Burla *et al.*, 2007 ▸), *SHELXL2014* (Sheldrick, 2015 ▸) and *OLEX2* (Dolomanov *et al.*, 2009 ▸).

## supplementary crystallographic information

### Crystal data

**Table:**

[Co(C_6_H_12_NO_4_)Cl]	*D*_x_ = 1.818 Mg m^−^^3^
*M**_r_* = 256.55	Mo *K*α radiation, λ = 0.71073 Å
Orthorhombic, *P**b**c**a*	Cell parameters from 9897 reflections
*a* = 8.3925 (9) Å	θ = 2.6–26.4°
*b* = 14.0939 (15) Å	µ = 2.10 mm^−^^1^
*c* = 15.8448 (17) Å	*T* = 296 K
*V* = 1874.2 (3) Å^3^	Prism, purple
*Z* = 8	0.84 × 0.27 × 0.24 mm
*F*(000) = 1048	

### Data collection

**Table:**

Bruker APEXII CCD diffractometer	1854 reflections with *I* > 2σ(*I*)
Graphite monochromator	*R*_int_ = 0.021
φ and ω scans	θ_max_ = 26.4°, θ_min_ = 2.6°
Absorption correction: multi-scan (SADABS; Bruker, 2004)	*h* = −10→10
*T*_min_ = 0.512, *T*_max_ = 0.604	*k* = −17→17
15130 measured reflections	*l* = −19→19
1933 independent reflections	

### Refinement

**Table:**

Refinement on *F*^2^	Primary atom site location: structure-invariant direct methods
Least-squares matrix: full	Hydrogen site location: mixed
*R*[*F*^2^ > 2σ(*F*^2^)] = 0.025	H atoms treated by a mixture of independent and constrained refinement
*wR*(*F*^2^) = 0.066	*w* = 1/[σ^2^(*F*_o_^2^) + (0.030*P*)^2^ + 1.540*P*] where *P* = (*F*_o_^2^ + 2*F*_c_^2^)/3
*S* = 1.14	(Δ/σ)_max_ = 0.001
1933 reflections	Δρ_max_ = 0.51 e Å^−^^3^
125 parameters	Δρ_min_ = −0.43 e Å^−^^3^
3 restraints	

### Special details

**Table:**

Geometry. All esds (except the esd in the dihedral angle between two l.s. planes) are estimated using the full covariance matrix. The cell esds are taken into account individually in the estimation of esds in distances, angles and torsion angles; correlations between esds in cell parameters are only used when they are defined by crystal symmetry. An approximate (isotropic) treatment of cell esds is used for estimating esds involving l.s. planes.

### Fractional atomic coordinates and isotropic or equivalent isotropic displacement parameters (Å^2^)

**Table:**

	*x*	*y*	*z*	*U*_iso_*/*U*_eq_	
Co1	0.41813 (3)	0.62528 (2)	0.09419 (2)	0.02152 (10)	
Cl1	0.15183 (6)	0.60446 (4)	0.07726 (4)	0.04084 (15)	
O1	0.46124 (15)	0.51884 (10)	0.17919 (8)	0.0254 (3)	
O2	0.42621 (16)	0.75436 (10)	0.15315 (9)	0.0315 (3)	
H2	0.3575 (17)	0.7980 (10)	0.1454 (15)	0.047*	
O3	0.48651 (16)	0.61778 (10)	−0.02717 (8)	0.0320 (3)	
O4	0.68239 (18)	0.63508 (9)	−0.11936 (9)	0.0305 (3)	
N1	0.67257 (18)	0.64315 (11)	0.10881 (9)	0.0226 (3)	
C1	0.6259 (2)	0.50535 (15)	0.20204 (13)	0.0319 (4)	
H1A	0.6481	0.4381	0.2078	0.038*	
H1B	0.6469	0.5356	0.2559	0.038*	
C2	0.7323 (2)	0.54745 (14)	0.13522 (13)	0.0308 (4)	
H2A	0.8398	0.5533	0.1571	0.037*	
H2B	0.7353	0.5056	0.0866	0.037*	
C3	0.5816 (2)	0.79707 (15)	0.15957 (15)	0.0367 (5)	
H3A	0.5852	0.8407	0.2069	0.044*	
H3B	0.6066	0.8318	0.1084	0.044*	
C4	0.6996 (2)	0.71678 (15)	0.17250 (13)	0.0336 (4)	
H4A	0.8075	0.7408	0.1677	0.040*	
H4B	0.6865	0.6901	0.2285	0.040*	
C5	0.6287 (2)	0.63779 (12)	−0.04522 (12)	0.0240 (4)	
C6	0.7392 (2)	0.66736 (15)	0.02554 (11)	0.0301 (4)	
H6A	0.8412	0.6359	0.0186	0.036*	
H6B	0.7571	0.7353	0.0226	0.036*	
H1	0.432 (3)	0.468 (2)	0.1653 (18)	0.054 (9)*	

### Atomic displacement parameters (Å^2^)

**Table:**

	*U*^11^	*U*^22^	*U*^33^	*U*^12^	*U*^13^	*U*^23^
Co1	0.01510 (14)	0.02795 (15)	0.02151 (15)	−0.00134 (9)	−0.00195 (9)	0.00217 (9)
Cl1	0.0166 (2)	0.0604 (3)	0.0455 (3)	−0.0060 (2)	−0.0050 (2)	0.0169 (3)
O1	0.0233 (6)	0.0265 (7)	0.0265 (7)	−0.0020 (5)	−0.0014 (5)	0.0011 (5)
O2	0.0269 (7)	0.0294 (7)	0.0383 (8)	0.0047 (5)	−0.0040 (6)	−0.0023 (6)
O3	0.0217 (7)	0.0523 (9)	0.0220 (7)	−0.0095 (6)	−0.0014 (5)	−0.0024 (6)
O4	0.0336 (7)	0.0355 (7)	0.0223 (7)	−0.0109 (6)	0.0038 (6)	−0.0020 (5)
N1	0.0169 (7)	0.0310 (8)	0.0199 (7)	−0.0025 (6)	−0.0026 (6)	0.0015 (6)
C1	0.0263 (9)	0.0363 (10)	0.0330 (10)	0.0024 (8)	−0.0079 (8)	0.0062 (8)
C2	0.0228 (9)	0.0359 (10)	0.0337 (10)	0.0036 (8)	−0.0030 (8)	0.0022 (8)
C3	0.0367 (11)	0.0290 (10)	0.0445 (12)	−0.0038 (8)	−0.0073 (9)	−0.0057 (9)
C4	0.0280 (10)	0.0406 (11)	0.0323 (10)	−0.0047 (8)	−0.0069 (8)	−0.0066 (8)
C5	0.0247 (8)	0.0235 (8)	0.0238 (9)	−0.0031 (7)	−0.0006 (7)	0.0009 (7)
C6	0.0217 (8)	0.0443 (11)	0.0243 (9)	−0.0090 (8)	−0.0001 (7)	0.0027 (8)

### Geometric parameters (Å, º)

**Table:**

Co1—Cl1	2.2701 (6)	C1—H1A	0.9700
Co1—O1	2.0482 (13)	C1—H1B	0.9700
Co1—O2	2.0463 (14)	C1—C2	1.507 (3)
Co1—O3	2.0095 (14)	C2—H2A	0.9700
Co1—N1	2.1626 (15)	C2—H2B	0.9700
O1—C1	1.441 (2)	C3—H3A	0.9700
O1—H1	0.79 (3)	C3—H3B	0.9700
O2—H2	0.852 (9)	C3—C4	1.518 (3)
O2—C3	1.440 (2)	C4—H4A	0.9700
O3—C5	1.259 (2)	C4—H4B	0.9700
O4—C5	1.259 (2)	C5—C6	1.514 (3)
N1—C2	1.498 (2)	C6—H6A	0.9700
N1—C4	1.465 (2)	C6—H6B	0.9700
N1—C6	1.473 (2)		
			
O1—Co1—Cl1	99.03 (4)	C2—C1—H1B	109.7
O1—Co1—N1	80.82 (6)	N1—C2—C1	110.66 (16)
O2—Co1—Cl1	101.63 (4)	N1—C2—H2A	109.5
O2—Co1—O1	110.19 (6)	N1—C2—H2B	109.5
O2—Co1—N1	79.33 (6)	C1—C2—H2A	109.5
O3—Co1—Cl1	99.28 (4)	C1—C2—H2B	109.5
O3—Co1—O1	122.70 (6)	H2A—C2—H2B	108.1
O3—Co1—O2	118.31 (6)	O2—C3—H3A	110.4
O3—Co1—N1	80.02 (6)	O2—C3—H3B	110.4
N1—Co1—Cl1	179.02 (5)	O2—C3—C4	106.80 (16)
Co1—O1—H1	115 (2)	H3A—C3—H3B	108.6
C1—O1—Co1	115.54 (11)	C4—C3—H3A	110.4
C1—O1—H1	104 (2)	C4—C3—H3B	110.4
Co1—O2—H2	123.6 (13)	N1—C4—C3	109.51 (16)
C3—O2—Co1	115.72 (12)	N1—C4—H4A	109.8
C3—O2—H2	108.8 (12)	N1—C4—H4B	109.8
C5—O3—Co1	118.44 (12)	C3—C4—H4A	109.8
C2—N1—Co1	104.78 (11)	C3—C4—H4B	109.8
C4—N1—Co1	108.01 (12)	H4A—C4—H4B	108.2
C4—N1—C2	113.16 (15)	O3—C5—C6	118.30 (16)
C4—N1—C6	113.21 (15)	O4—C5—O3	123.00 (17)
C6—N1—Co1	107.81 (11)	O4—C5—C6	118.69 (17)
C6—N1—C2	109.38 (15)	N1—C6—C5	111.54 (15)
O1—C1—H1A	109.7	N1—C6—H6A	109.3
O1—C1—H1B	109.7	N1—C6—H6B	109.3
O1—C1—C2	109.85 (15)	C5—C6—H6A	109.3
H1A—C1—H1B	108.2	C5—C6—H6B	109.3
C2—C1—H1A	109.7	H6A—C6—H6B	108.0
			
Co1—O1—C1—C2	−21.0 (2)	O3—C5—C6—N1	15.5 (3)
Co1—O2—C3—C4	−35.2 (2)	O4—C5—C6—N1	−165.10 (16)
Co1—O3—C5—O4	179.97 (13)	C2—N1—C4—C3	−156.64 (17)
Co1—O3—C5—C6	−0.7 (2)	C2—N1—C6—C5	92.75 (18)
Co1—N1—C2—C1	−44.47 (17)	C4—N1—C2—C1	73.0 (2)
Co1—N1—C4—C3	−41.12 (19)	C4—N1—C6—C5	−140.06 (17)
Co1—N1—C6—C5	−20.64 (19)	C6—N1—C2—C1	−159.82 (15)
O1—C1—C2—N1	44.4 (2)	C6—N1—C4—C3	78.2 (2)
O2—C3—C4—N1	49.9 (2)		

### Hydrogen-bond geometry (Å, º)

**Table:**

*D*—H···*A*	*D*—H	H···*A*	*D*···*A*	*D*—H···*A*
O2—H2···O4^i^	0.85 (1)	1.79 (1)	2.6271 (19)	165 (2)
O1—H1···O4^ii^	0.79 (3)	1.89 (3)	2.6567 (19)	165 (3)

Symmetry codes: (i) *x*−1/2, −*y*+3/2, −*z*; (ii) −*x*+1, −*y*+1, −*z*.
