# Supplementary figures and images for: Crystal structure, solvothermal synthesis, thermogravimetric studies and DFT calculations of a five-coordinate cobalt(II) compound based on the N,N-bis­(2-hy­droxy­eth­yl)glycine anion
Source: Acta Crystallogr E Crystallogr Commun. 2016 Sep 23;72(Pt 10):1463–7. doi: 10.1107/S2056989016014596 (PMC5050777; doi:10.1107/S2056989016014596)

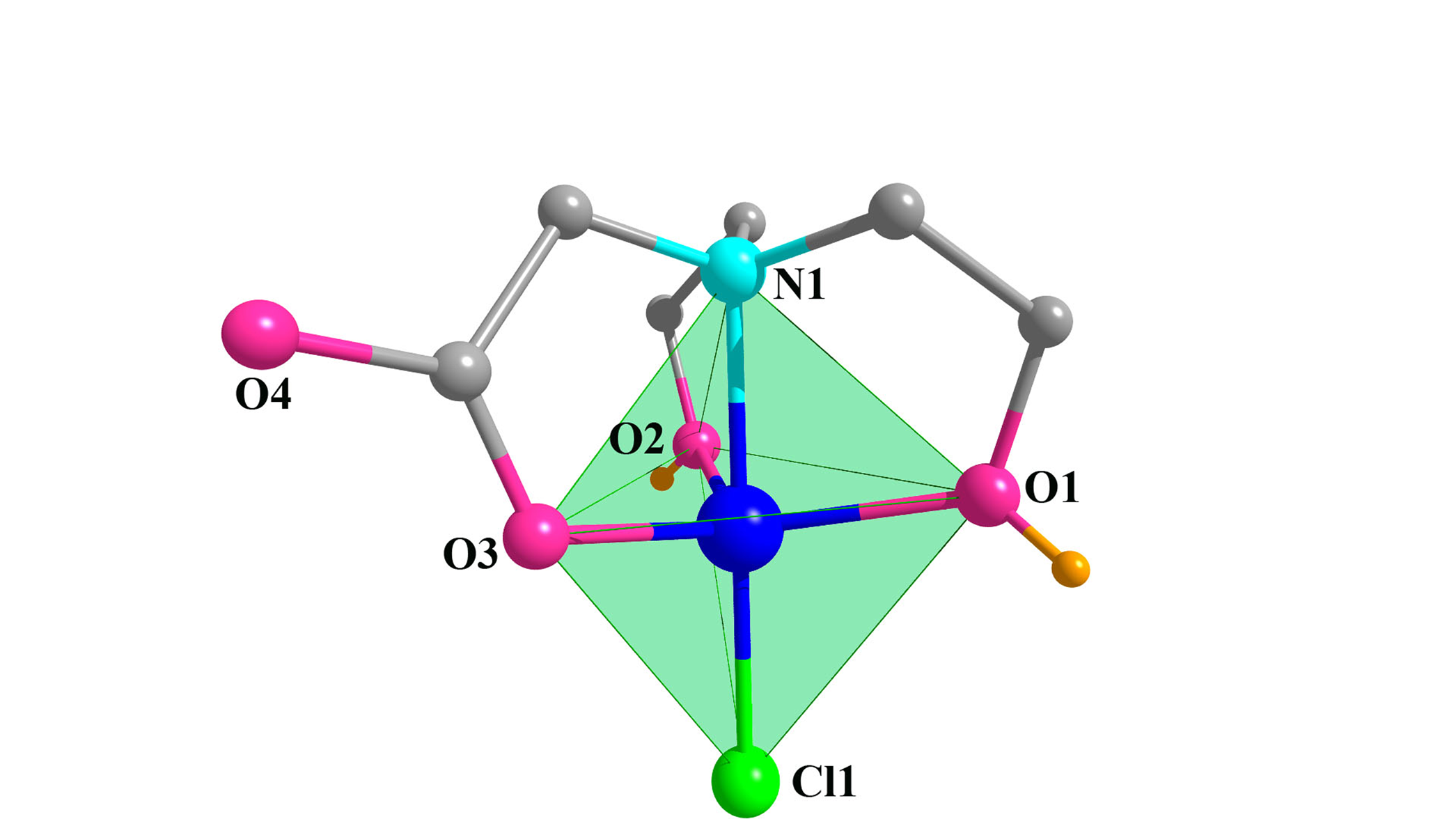

Supplement: Supplementary file 3 [file e-72-01463-sup3.tif]
