# Supplementary material for: Crystal structure, solvothermal synthesis, thermogravimetric studies and DFT calculations of a five-coordinate cobalt(II) compound based on the N,N-bis­(2-hy­droxy­eth­yl)glycine anion
Source: Acta Crystallogr E Crystallogr Commun. 2016 Sep 23;72(Pt 10):1463–7. doi: 10.1107/S2056989016014596 (PMC5050777; doi:10.1107/S2056989016014596)
Supplement: Supplementary file 4 [file e-72-01463-sup4.pdf]

Supporting information

Crystal structure, solvothermal synthesis, and thermogravimetric studies of a five-coordinated cobalt(II) compound based on the anion of *N*,*N*-bis(2-hydroxyethyl)glycine

Yanling Zhou, Xianrong Liu, Qijun Wang, Lisheng Wang, Baoling Song

1. Cartesian coordinates of the structures reported in the paper with the calculated (U)B3LYP electronic energy (hartrees).

(*S* =1/2) -1196.84535869					(*S* =3/2) -1196.86664506				
1	Co	-0.61005300	-0.27353300	0.19072900	1	Co	0.79321700	0.31825100	0.09806800
2	Cl	-2.56449100	-1.40277100	0.06178900	2	Cl	3.00650100	0.84160400	-0.09523500
3	O	0.04257900	0.45282100	2.17906200	3	O	0.45940200	-0.34979800	2.12115000
4	H	0.53313700	-0.35992300	2.40439900	4	H	0.27550900	0.44653600	2.64859900
5	O	-1.68198800	1.36763700	-0.35851900	5	O	1.18314400	-1.43190100	-1.07904100
6	H	-2.54094800	0.91445400	-0.52133600	6	H	1.96522100	-1.20925500	-1.61657700
7	O	0.64095800	-1.69032200	0.32298700	7	O	-0.37464300	1.83748300	-0.15562300
8	O	2.56530300	-2.36442800	-0.65064900	8	O	-2.46951200	2.60879100	-0.50139900
9	N	0.97767500	0.81235200	-0.46742400	9	N	-1.25644700	-0.72595500	-0.12033300
10	C	0.96183300	1.51962100	1.92122000	10	C	-0.63880700	-1.27930100	2.21653400
11	H	1.60511100	1.70180400	2.79087200	11	H	-1.00573900	-1.32355300	3.24752100
12	H	0.33469100	2.40277100	1.77317700 13 C	12	H	-0.20378800	-2.25109700	1.97060000
		1.82135500	1.22092200	0.69144600	13	C	-1.76359900	-0.89830200	1.25642000
14	H	2.44757500	2.08611600	0.43284900	14	H	-2.57135600	-1.64603300	1.30767700
15	H	2.49421800	0.38901000	0.91765700	15	H	-2.19072200	0.05957200	1.57111900
16	C	-1.02941300	1.73476200	-1.59538900	16	C	0.06194400	-1.88733300	-1.84931100
17	H	-1.48135500	2.64554200	-2.00361700	17	H	0.27524200	-2.86613800	-2.29395000
18	H	-1.13412000	0.92453000	-2.32761500	18	H	-0.16549800	-1.17608600	-2.65314900
19	C	0.43344100	1.97603600	-1.22658300	19	C	-1.08675900	-1.99860500	-0.84979200
20	H	1.03352600	2.18092300	-2.12200500	20	H	-2.01138700	-2.30519700	-1.36110500
21	H	0.48468000	2.85789700	-0.58145400	21	H	-0.83080000	-2.78221300	-0.13000400
22	C	1.66380300	-1.56313400	-0.48677700	22	C	-1.63570600	1.71951400	-0.46401100
23	C	1.66785200	-0.22558300	-1.28390800	23	C	-2.05043800	0.27941900	-0.86205400
24	H	2.69319100	0.06719200	-1.53950600	24	H	-3.12888200	0.14289300	-0.71223100
25	H	1.10630000	-0.36567500	-2.21466300	25	H	-1.84698600	0.16324800	-1.93240600

2. The coordination distances (Co-X, Å) of two calculated structures are presented.

S = 1/2		*S* = 3/2	
Bond	Length (Å)	Bond	Length(Å)
Co-O(1)	1.89468	Co-O(1)	1.93296
Co-O(2)	2.21517	Co-O(2)	2.15652
Co-O(3)	2.03572	Co-O(3)	2.14492
Co-Cl	2.26089	Co-Cl	2.28252
Co-N	2.03303	Co-N	2.31067
